# Shoulder girdle resection: surgical technique modification and introduction of a new classification system

**DOI:** 10.1186/s12957-019-1636-2

**Published:** 2019-06-18

**Authors:** Ahmad Shehadeh, Ahmad Ja’afar, Ula Isleem, Anas Hamad, Ahmed Salem

**Affiliations:** 1 Department of Surgical Oncology, Orthopedic Oncology Unit, King Hussein Cancer Center, Queen Rania Al Abdullah Street, P.O. Box 1269, Amman, 11941 Jordan; 2 Faculty of Medicine, University of Jordan, Amman, Jordan; 3 Division of Clinical Oncology, The University of Manchester, Manchester, UK

**Keywords:** Surgical technique, Orthopedics, Tumor resection, Classification system

## Abstract

**Objective:**

Different classification systems for surgical tumor resections in the proximal humerus and scapula have been described, but none are specific or have been recently revised. The purpose of this article is to report modified surgical techniques and a new classification system for resections in the humerus and scapula.

**Methods:**

Thirty-two patients with shoulder girdle bone tumors were operated upon. Two separate new classifications were assigned to resections in the humerus (types I–IV) and scapula (types I–III). An annotation is added to signify deltoid preservation (A) or sacrifice (B). Modified surgical techniques were devised.

**Results:**

For extra-articular resections of the proximal humerus, we show that sacrificing the acromion and coracoid process is not required. Preservation of these structures can improve cosmetic shoulder outcome. For tumors with no large medial component, we show that there is no need to detach the muscle attachment from the coracoid process allowing earlier elbow extension postoperatively. After a mean follow-up period of 46 months, only two patients developed local recurrence. Postoperative infection was seen in two and stem loosening in one patient. The average MSTS functional score for all patients was 83%.

**Conclusion:**

Our modified surgical techniques saved structures which were unnecessarily resected with no advantage in surgical series. We reserved the integrity of more muscular tissues and attachments leading to less restriction during the rehabilitation process. This new classification system is realistic, easy to implement, and applicable to all patients.

## Introduction

Shoulder girdle resection and reconstruction are some of the most demanding surgeries in the field of orthopedic oncology. Due to the proximity of neurovascular structures such as the brachial plexus and the axillary vessels, meticulous surgical techniques and skills are essential. More than 95% of patients with shoulder sarcomas can be safely treated by limb-sparing surgical techniques [1]. For a successful outcome, attention should be paid to the skeletal and muscular reconstruction of the surgical defect [2]. A few published reports have reviewed and proposed surgical techniques and classifications for shoulder girdle tumor resection [2, 3].

In a study involving 38 patients with shoulder girdle tumors (92% were malignant, average follow-up was 4.6 years), the Malawer et al classified surgical techniques into six categories based on the structures removed, relation to the glenohumeral joint, and the status of the abductor mechanism. All of the operations fit this classification system [2]. In another article from Mayo Clinic, 57 patients with shoulder girdle tumors underwent limb-sparing surgeries and were assessed after an average of 5.3 years for intermediate functional results. Results and complications were related to the type of resection, reconstruction (spacers, osseous arthrodesis, and proximal humeral prosthesis), and the patients’ needs [3].

However, these two classifications do not differentiate between soft tissue and bone sarcomas. Also, proximal humeral resections are not separately appraised from scapular resections given the differences in the surgical approach and reconstruction method as is the case in other body sites (e.g., proximal femur vs*.* pelvic resections).

There is, therefore, an unmet need to revise these surgical classifications and report modified surgical techniques to allow optimal oncological and functional outcomes. Here, we report modified surgical techniques and a new classification system for resections in the humerus and scapula.

## Materials and methods

This study was performed in the King Hussein Cancer Center, which is the sole comprehensive cancer center in Jordan. The first author (ASh) proposed and implemented modified humerus and scapula surgical resection techniques with the aim of improving functional and cosmetic patient outcomes. In addition, a new classification system for these surgical procedures was proposed.

Between 2006 and 2015, AS performed 32 shoulder girdle resections (humerus (*n* = 26) and scapula (*n* = 6)) for tumor lesions. The histological subtypes of the resected tumors were Ewing sarcoma (*n* = 11), osteosarcoma (*n* = 9), metastasis (*n* = 5), and other tumors (*n* = 7). The median patient age was 19 years (range, 9–60) with 14 males and 18 females.

### Shehadeh classification

**Fig. 1:**
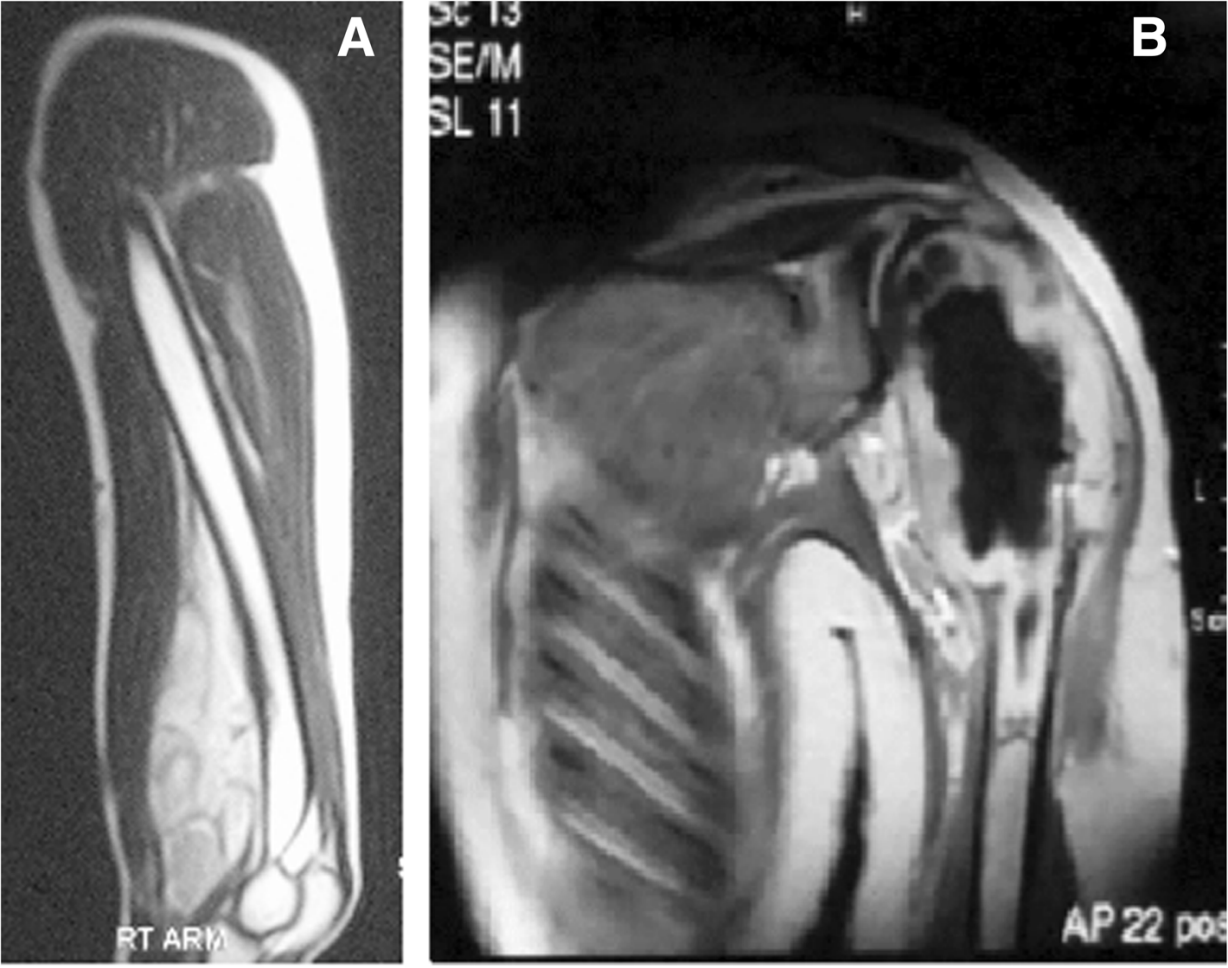
Soft tissue sarcoma abutting the bone (**a**) is approached differently from Bone sarcoma with soft tissue component (**b**)

Resection of a bone sarcoma with a soft tissue component usually includes resecting the bone and the whole soft tissue, while in soft tissue sarcoma abutting the bone we resect the soft tissue alone and preserve the bone. (Fig. 1a, b).

Contrary to older classifications, this new classification differentiates between proximal humeral and scapular resections for the first time. This separation is long overdue since the surgical approach and reconstruction principles are totally different in these 2 sites in similarity to pelvic and femoral resections which have always had separate classifications.

### Humeral resection

**Fig. 2:**
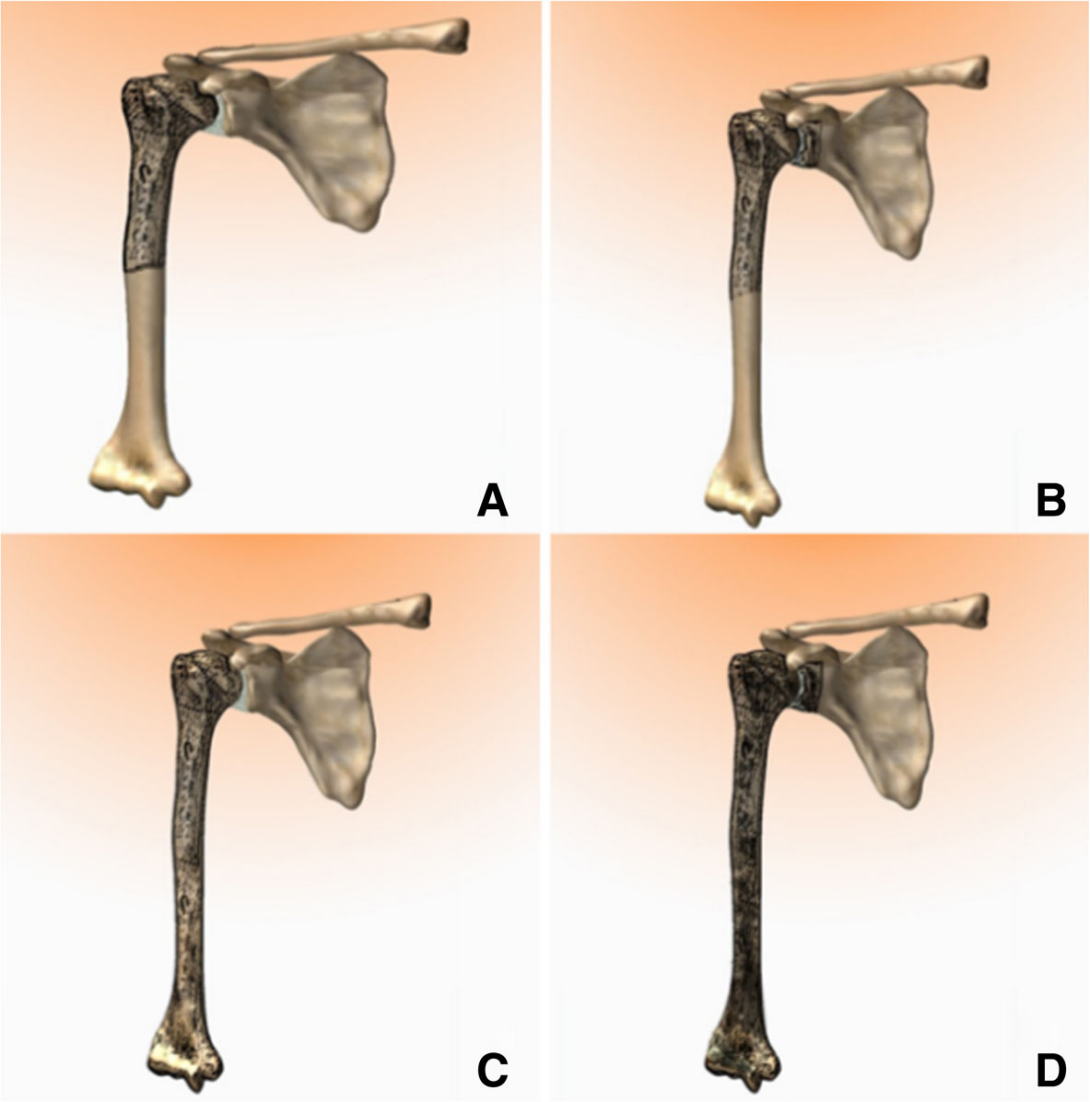
Types of humeral resection. **a** Type I—Intraarticular proximal humerus resection. **b** Type II—Extra-articular proximal humerus resection. **c** Type III—Intraarticular total humerus resection. **d** Type IV—Extra-articular total humerus resection

Resections of the humerus were classified into types I–IV (Fig. 2a, b, c, and d):

Type I: Intraarticular proximal humerus resection

Type II: Extra-articular proximal humerus resection

Type III: Intraarticular total humerus resection

Type IV: Extra-articular total humerus resection

Each type can be designated into A or B as follows:

A: Partial deltoid resection

B: Complete deltoid resection

### Scapular resection

**Fig. 3:**
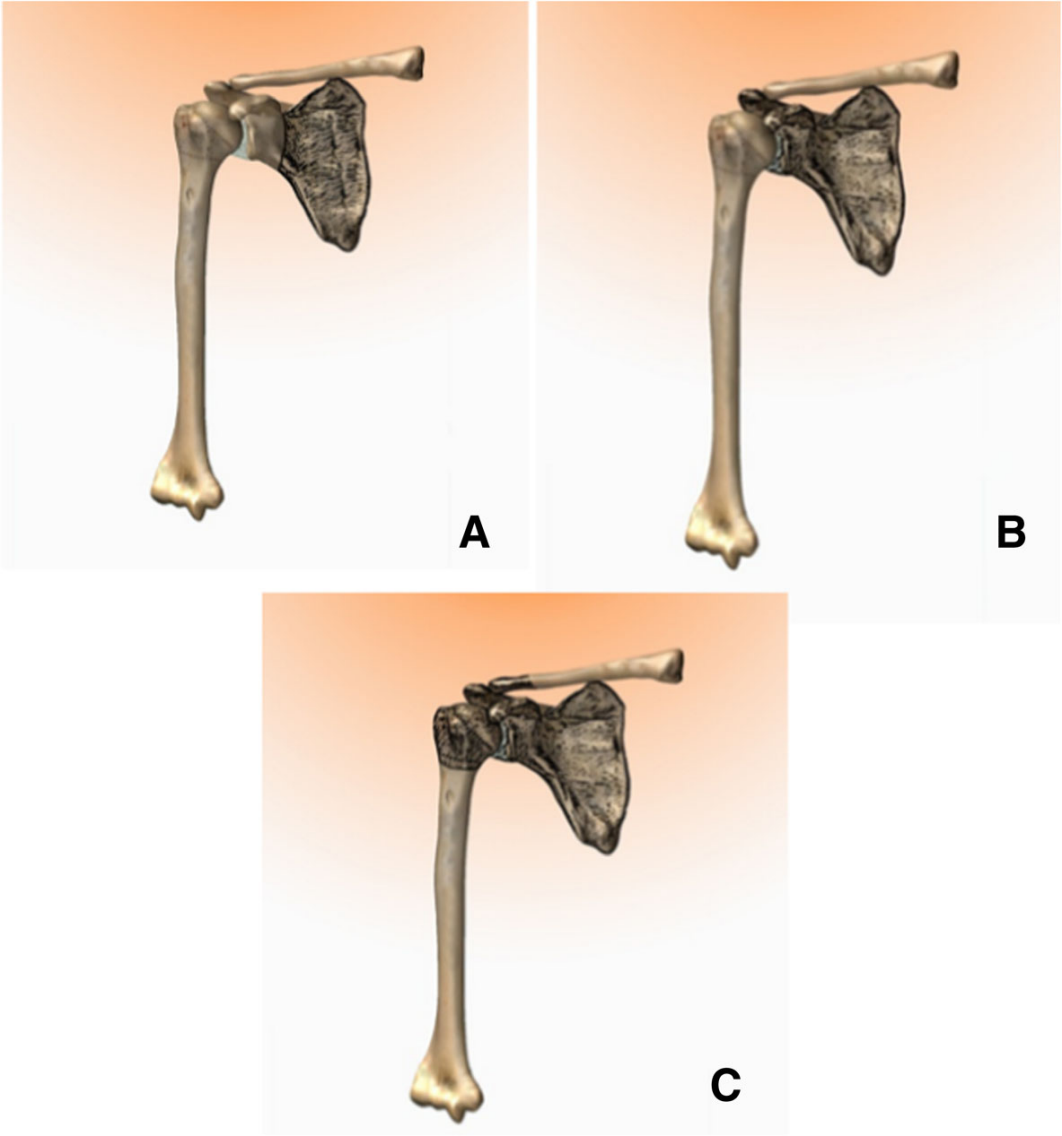
Resections of the scapula. **a** Type I—Partial scapular resection, with preservation of the glenoid and the glenohumeral joint. **b** Type II—Intraarticular resection of the scapula. **c** Type III—Extra-articular resection of the scapula, known as Tikhoff-Linberg procedure

Resections of the scapula were classified into types I–III (Fig. 3a, b, and c):

Type I: Partial scapular resection, with preservation of the glenoid and the glenohumeral joint

Type II: Intraarticular resection of the scapula

Type III: Extra-articular resection of the scapula, known as Tikhoff-Linberg procedure

### Exclusion criteria

The new classification was applied to tumors of bone but excluded soft tissue tumors, as resection of soft tissue tumors abutting bone do not normally involve whole bone resection.

### Surgical techniques

#### Humeral resection

**Fig. 4:**
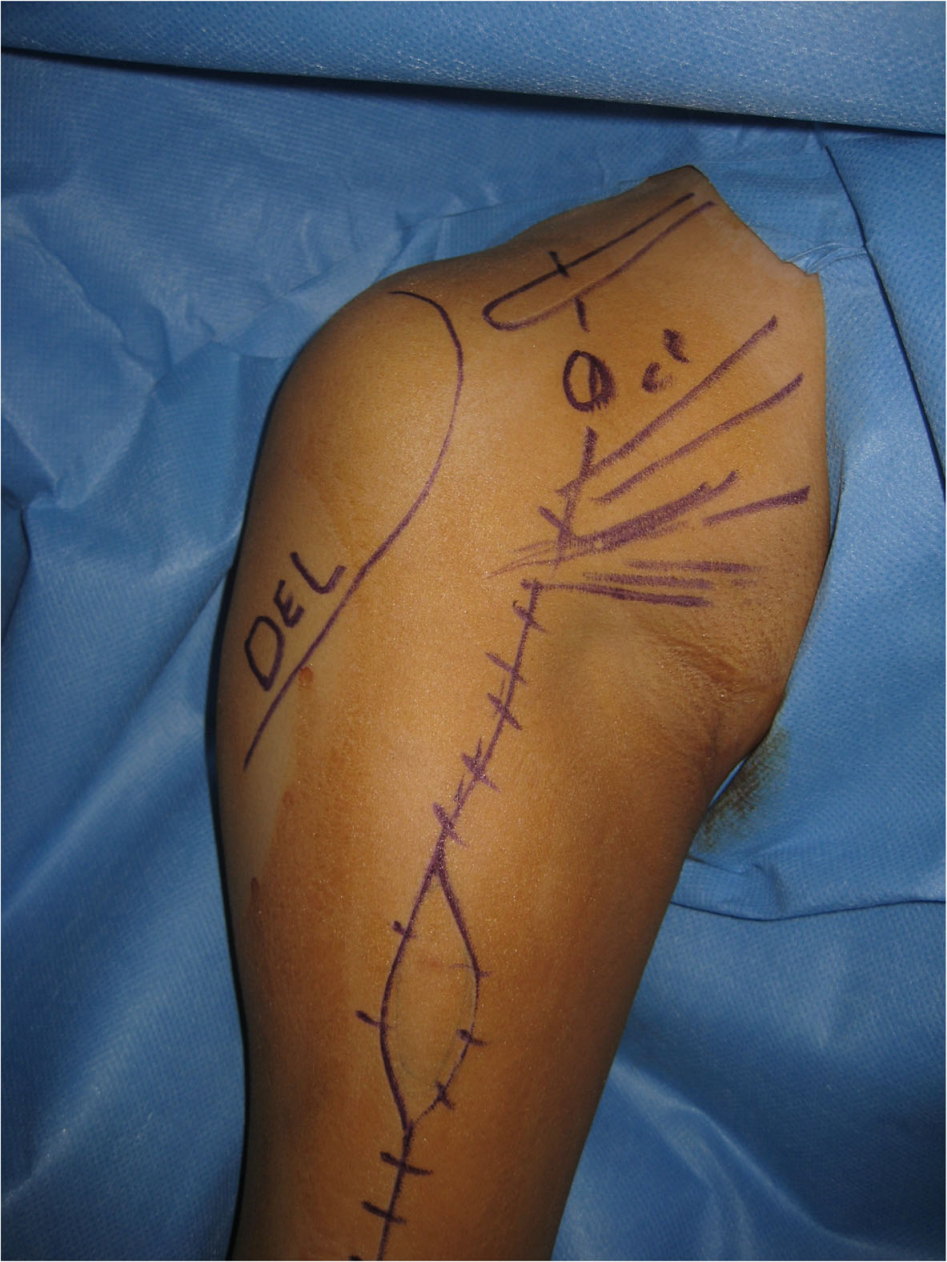
The Deltopectoral approach; starting from the lateral third of the clavicle and including all biopsy tracts

Here we describe a modified humeral resection approach. A deltopectoral approach is used; skin incision must include all the biopsy tract with the underlying segment of the deltoid muscle en bloc which is kept attached to the main specimen (Fig. 4).

**Fig. 5:**
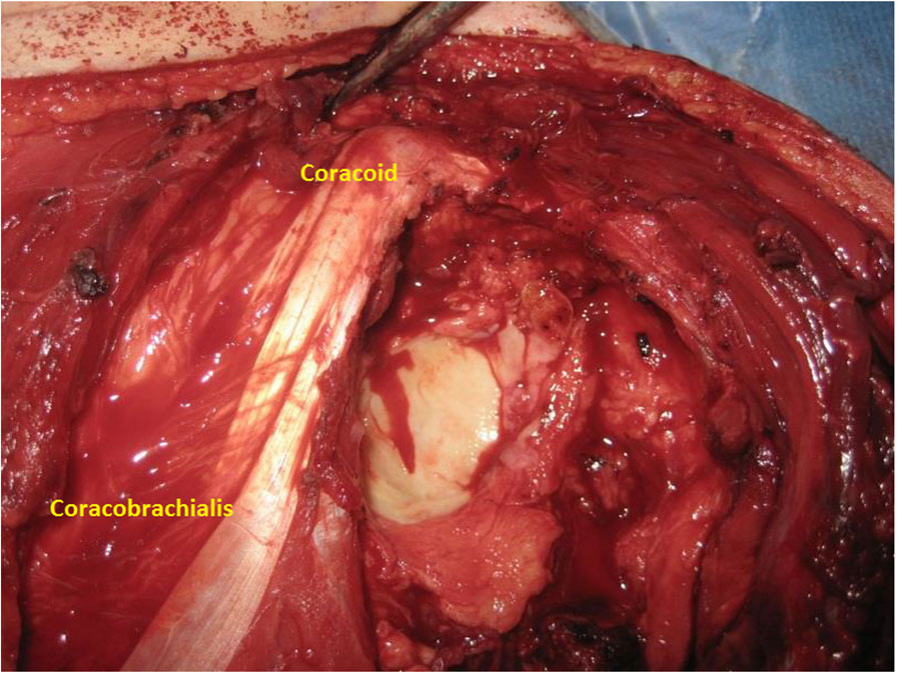
Conjoint tendon inserted into the coracoid process was preserved with no detachment

If the soft tissue component is large and stretching most of the deltoid muscle, then the deltoid sacrifice is indicated. If the soft tissue component is small or the tumor is completely intraosseous, then deltoid sparing can be achieved except for the part directly underneath the biopsy tract which should be excised en bloc with the main specimen. Medially, we identify and retract the conjoint tendon, with no need for release of the conjoint tendon from its insertion at the coracoid process (Fig. 5).

This is in variance to surgical techniques reported previously [4]. Then we identify and spare the axillary nerve followed by identification of the humeral circumflex vessels and transection. This is followed for types I and III with glenohumeral joint arthrotomy leaving part of the joint capsule for delayed reconstruction.

**Fig. 6:**
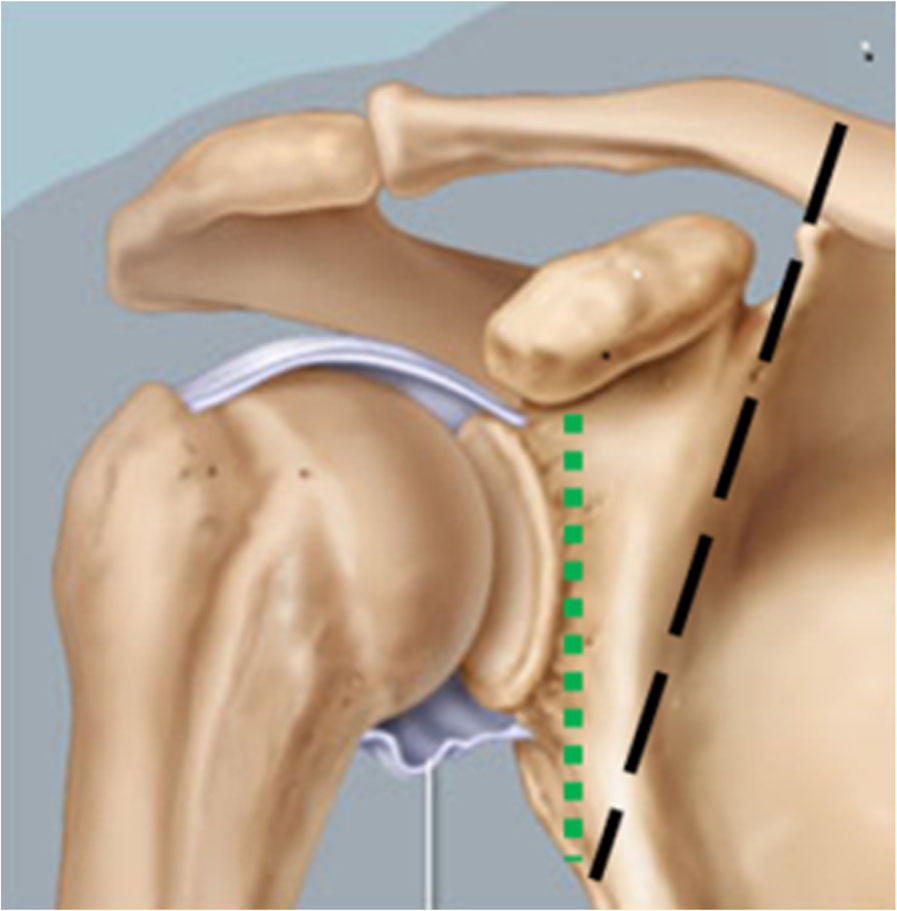
The green dotted line is the modified resection line by the author of this paper, while the black dashed line is the resection line used in previous publications

For types II and IV, we perform extra-articular resection just medial to the attachment of the capsule at the glenoid neck. This proposed modification is in contradiction to previous publications where the distal third of the clavicle and the whole acromion and coracoid process are sacrificed (Fig. 6).

**Fig. 7:**
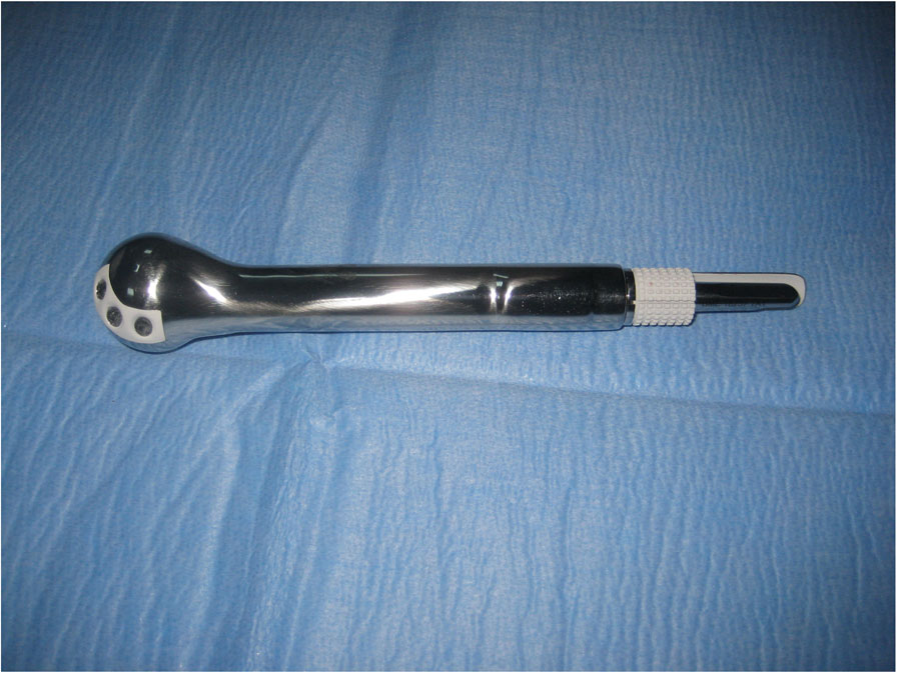
Tumor prosthesis for proximal humerus reconstruction

If reconstruction of the skeletal defect is to be contemplated via tumor prosthesis (Fig. 7), we prepare the humeral stump to insert the implant and fix it in the proper orientation. Cemented implants are preferred over cementless counterparts since many of those patients are also treated with chemotherapy which might affect osteointegration for cementless stem [2].

**Fig. 8:**
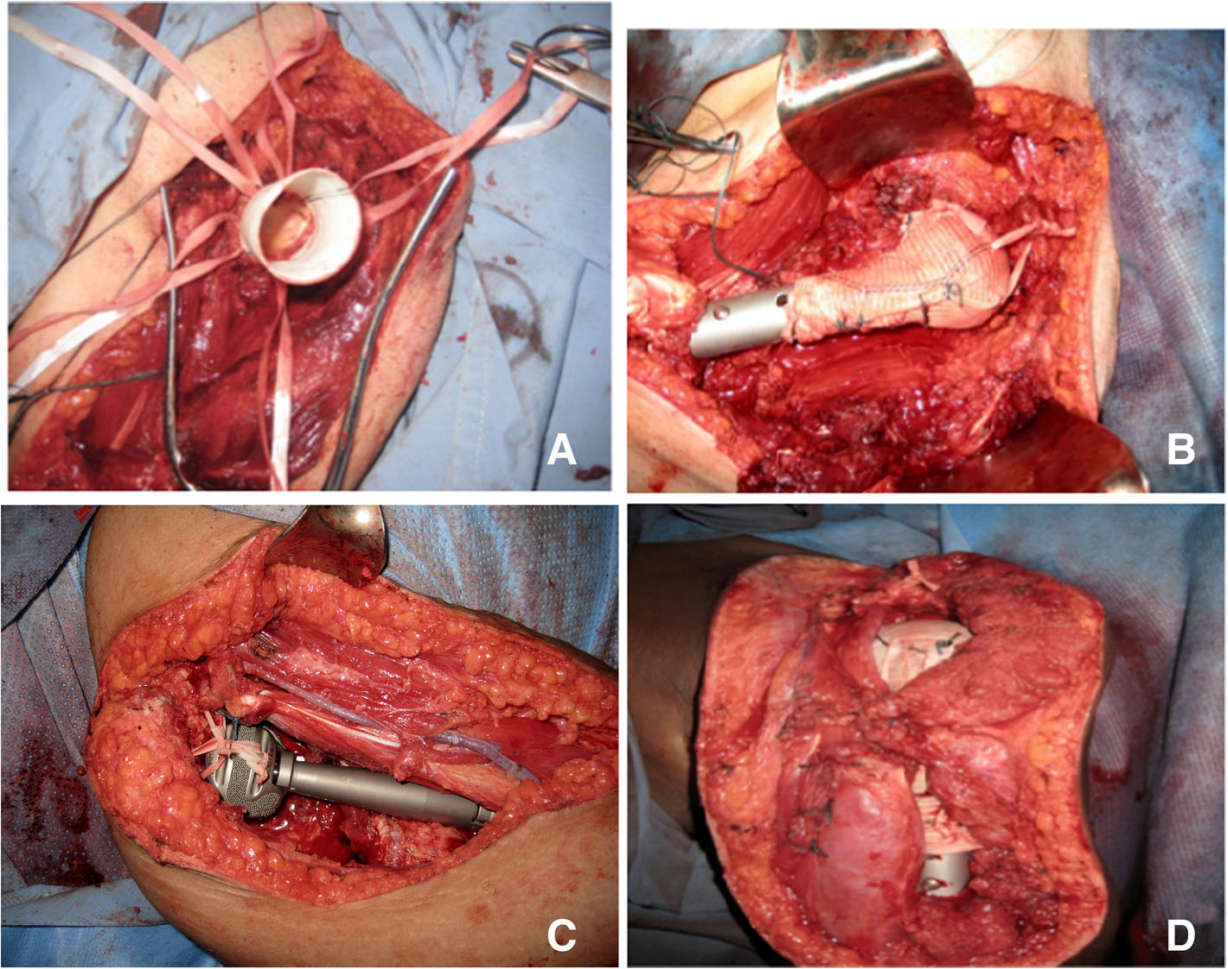
**a** The Goretex aortic graft attached to one side of the glenoid and then wrapped around the implant **b**. **c** In Extra-articular resection; Prosthetic head positioned in the space between the acromion and the coracoid process using a Dacron tape. **d** Reattachment of key muscle groups to the prosthesis through a Goretex sleeve

For joint reconstruction in types I and III, we use the Goretex aortic graft. This is attached in one side to the glenoid and then warped around the implant as described previously [3] (Fig. 8a, b).

For types II and IV, the prosthetic head is positioned in the space between and acromion and the coracoid using a Dacron tape (Fig. 8c). We re-attach the key muscle group to the prosthesis through a Goretex sleeve (Fig. 8d).

#### Scapula resection

**Fig. 9:**
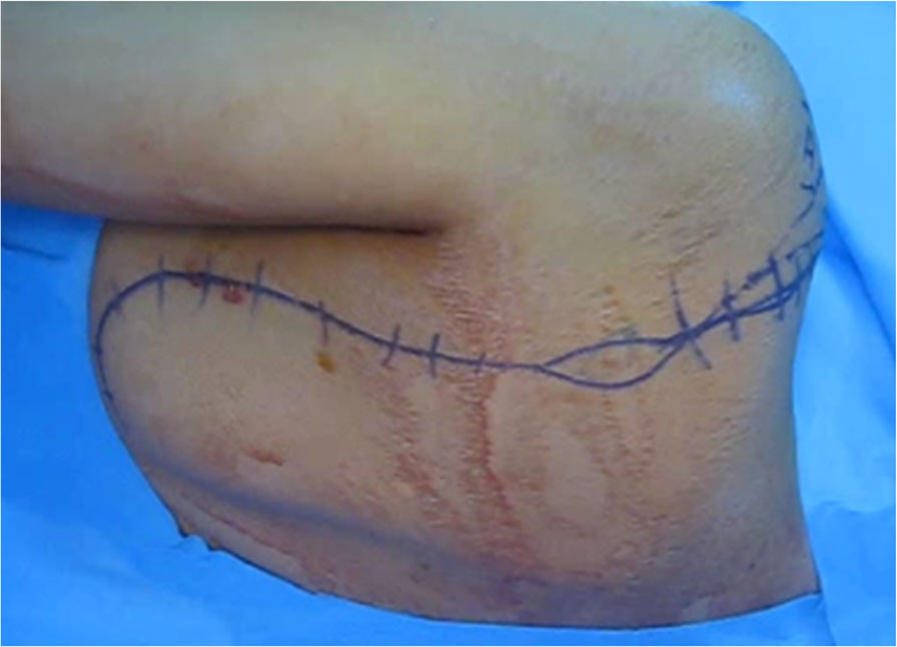
Incision line; at the lateral border of the scapula, including biopsy tract, and extending to the acromioclavicular joint

The patient is positioned in a flexible lateral position enabling the operators to flip the patient into the supine position when in need to allow anterior approach. An incision is created starting from the lateral border of the scapula, extending to acromioclavicular joint superiorly, and then proceeding to the coracoid anteriorly, as required (Fig. 9).

**Fig. 10:**
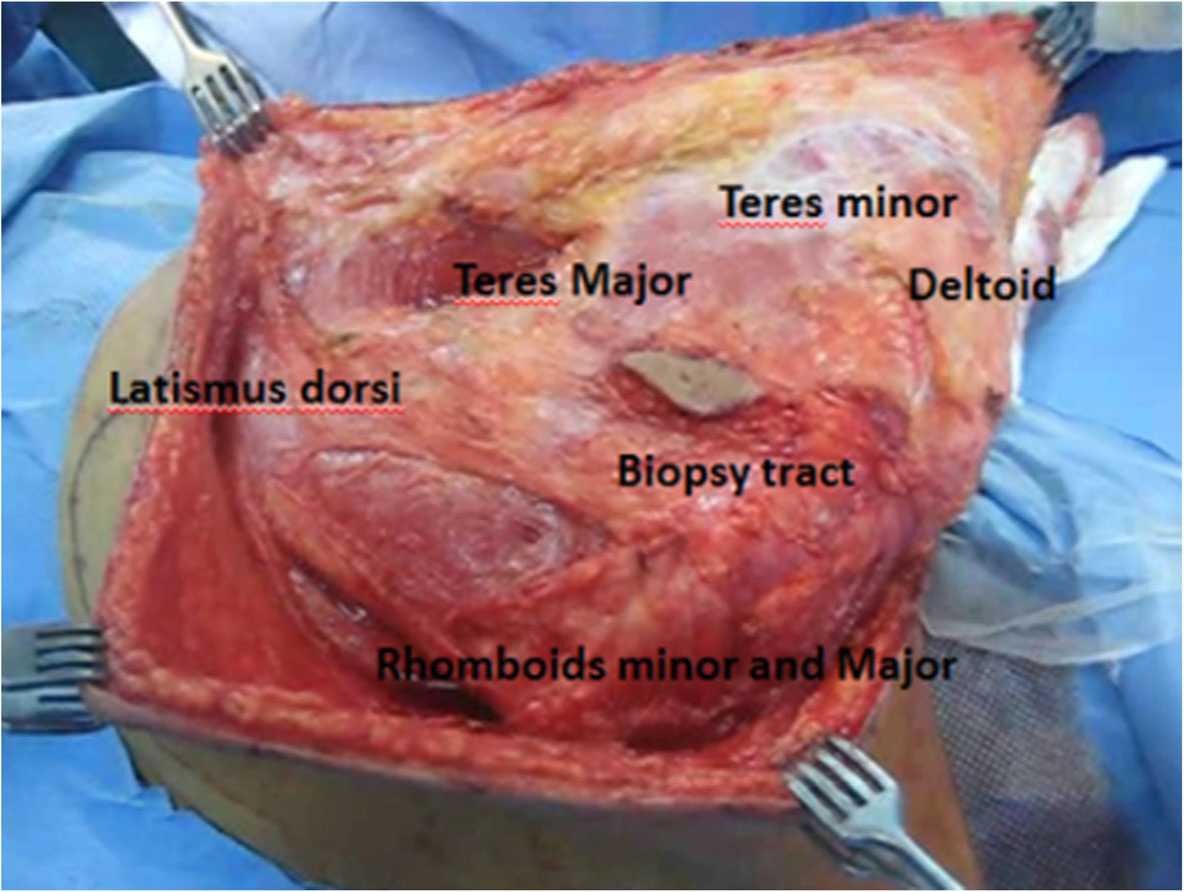
Key muscles attached to the scapula

The periscapular muscles are dissected off the lateral and medial borders of the scapula then elevating the scapula from the chest wall by detaching the serratus anterior muscle (Fig. 10).

For type II, we perform arthrotomy at the glenohumeral joint capsule and complete the release of the scapula intraarticularly.

**Fig. 11:**
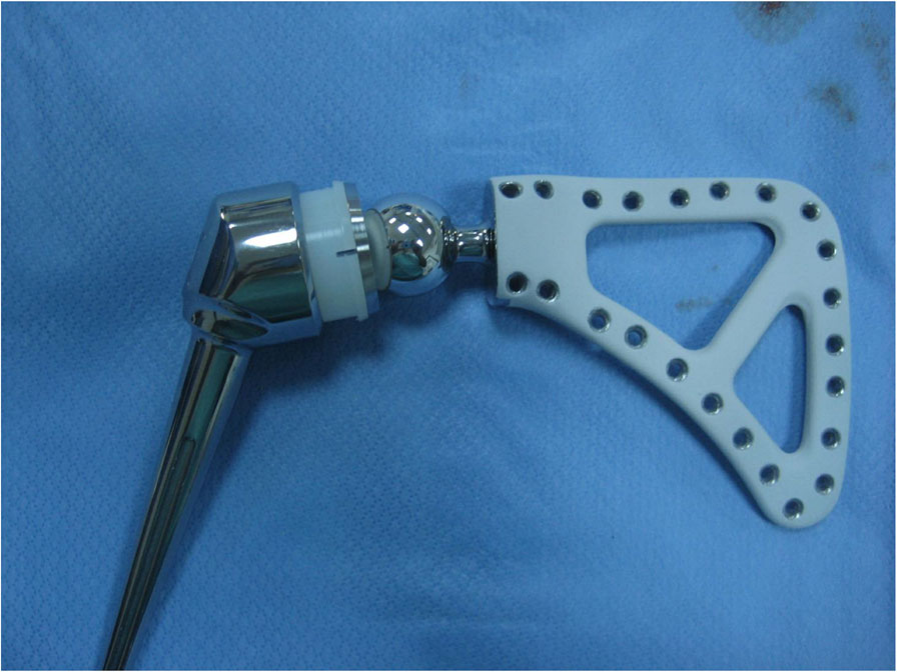
Custom-made scapular prosthesis

For type III, we resect the head of the humerus at the level of the anatomical neck and remove the specimen. For pediatric patients, we do not reconstruct, while for adults, we use a custom-made prosthesis (Fig. 11). This only slightly improves function; however, the cosmetic appearance is much better [2].

**Fig. 12:**
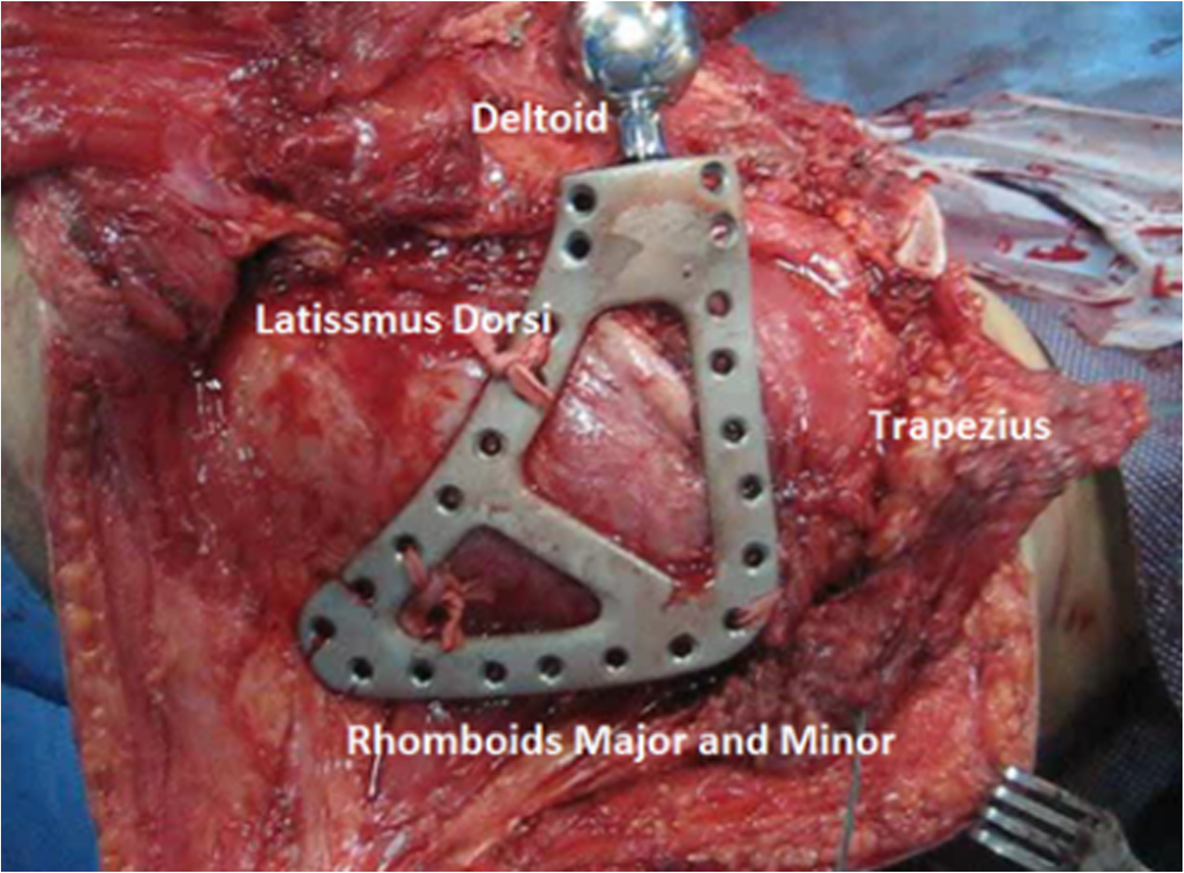
Scapular prosthesis attachment to the surrounding muscles

We attach the scapula to the serratus which acts as the bed for the implant and to the trapezius and rhomboids major and minor from the medial side and to the deltoid, teres major, and latissmus dorsi on the lateral side (Fig. 12).

**Fig. 13:**
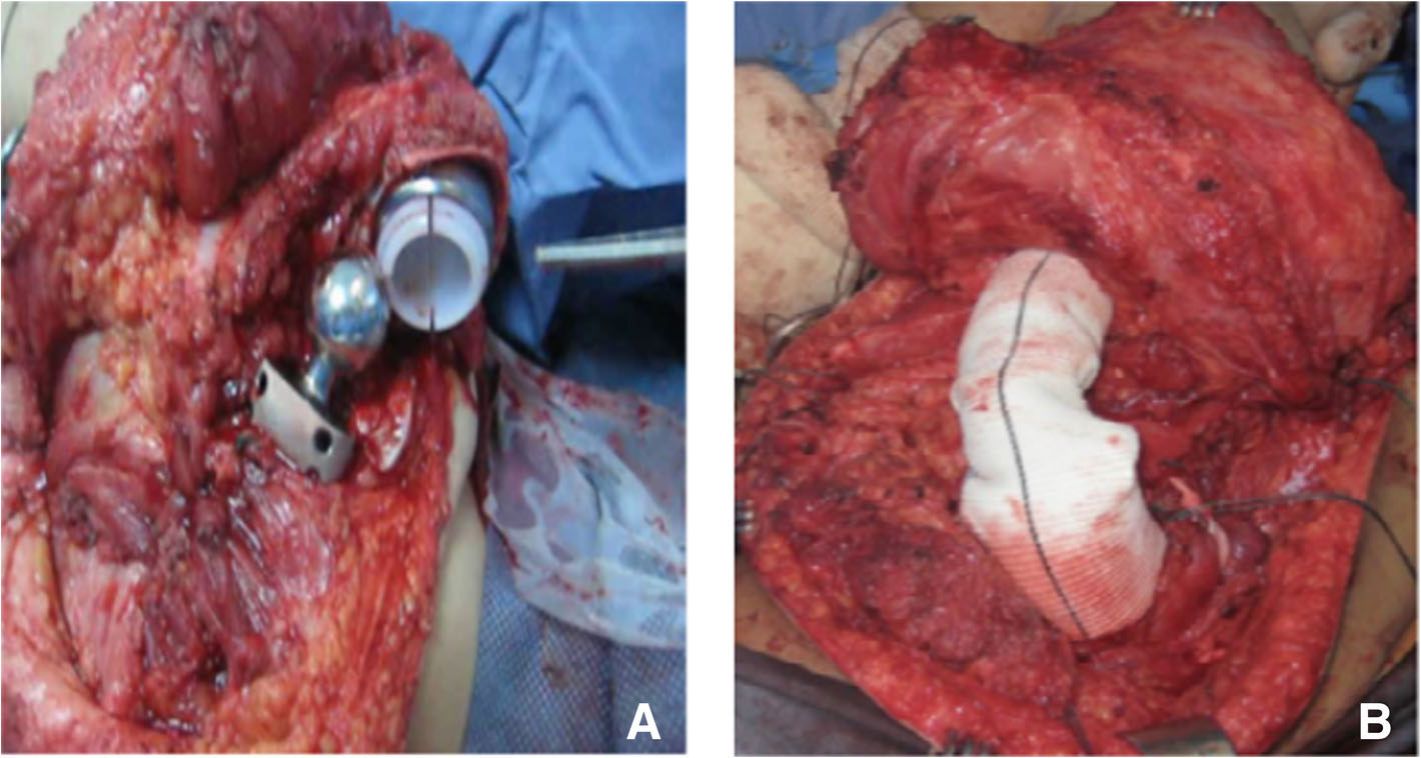
Constrained Glenohumueral joint before reduction (**a**), wrapping the Glenohumerual joint with Gortex graft for further reinforcemnt of stability (**b**)

We then insert the humeral component, using a Goretex graft to construct the capsule before reduction (Fig. 13a, b).

Then the field is closed in layers and a drain is inserted. All of our patients were offered standardized rehabilitation after limb salvage surgery [5].

## Results

A total of 32 patients were operated on between 2006 and 2015. These patients were followed up for a mean follow-up period of 46 months (range, 18–120). Twenty-six patients had proximal humerus tumors and six harbored scapula tumors. According to our new classification, 16 cases were of humerus IA, four were of humerus IIB, six were of scapula III, three were of humerus IIA, one was of humerus IIIA, one was of humerus IVA, and one was of humerus IB (Table 1).

**Table 1:** Resections of the scapula were classified into types I–III

Serial #	Age	Histological Diagnosis	Anatomical Location	Resection Type	Complications	Reconstruction Modality	Recurrence
1	12	Osteosarcoma	Left Humerus	Type IIB Humerus Resection	Superficial skin necrosis	Endoprosthesis	Yes
2	55	Metastatic Renal Cell Carcinoma	Left Proximal Humerus	Type IA Humerus Resection		Endoprosthesis	No
3	50	Chondrosarcoma	Left Proximal Humerus	Type IA Humerus Resection	Peri-prosthetic infection	Endoprosthesis	No
4	52	giant cell tumor	Left Proximal Humerus	Type IA Humerus Resection		Endoprosthesis	No
5	19	Gorham’s disease	Left Proximal Humerus	Type IA Humerus Resection	Failed allograft, revised with prosthesis	Osteoarticular Allograft	No
6	14	Osteosarcoma	Left Proximal Humerus	Type IIB Humerus Resection		Endoprosthesis	No
7	15	Ewing Sarcoma	Right Scapula	Type III Scapula Resection		Endoprosthesis/custom	No
8	17	Ewing Sarcoma	Right Proximal Humerus	Type IA Humerus Resection		Endoprosthesis	No
9	35	Recurrent Adamintinoma	Left Proximal Humerus	Type IA Humerus Resection		Osteoarticular Allograft	No
10	39	Metastatic Breast Cancer	Right Proximal Humerus	Type IA Humerus Resection		Endoprosthesis	No
11	60	Metastatic Parotid Cancer	Right Proximal Humerus	Type IA Humerus Resection		Endoprosthesis	No
12	5	Ewing Sarcoma	Right Scapula	Type III Scapula Resection		No reconstruction	No
13	7	Ewing Sarcoma	Right Scapula	Type III Scapula Resection		No reconstruction	No
14	18	Ewing Sarcoma	Left Proximal Humerus	Type IA Humerus Resection		Endoprosthesis	No
15	22	Aneurysmal Bone Cyst	Right Proximal Humerus	Type IB Humerus Resection		Endoprosthesis	No
16	10	Ewing Sarcoma	Left Total Humerus	Type IIIA Humerus Resection		Endoprosthesis	Yes
17	13	Ewing Sarcoma	Left Scapula	Type III Scapula Resection	Infection	Endoprosthesis/custom	No
18	55	GI metastasis	Left Proximal Humerus	Type IA Humerus Resection		Endoprosthesis	No
19	40	Giant Cell Tumor	Right Proximal Humerus	Type IA Humerus Resection		Endoprosthesis	No
20	19	Ewing Sarcoma	Left Proximal Humerus	Type IIA Humerus Resection		Endoprosthesis	No
21	16	Osteosarcoma	Right Proximal Humerus	Type IIA Humerus Resection		Endoprosthesis/custom	No
22	3	Ewing Sarcoma	Left Proximal Humerus	Type IA Humerus Resection		Osteoarticular Allograft	No
23	9	Osteosarcoma	Right Proximal Humerus	Type IIB Humerus Resection		Endoprosthesis/custom	No
24	12	Osteosarcoma	Left Whole Humerus	Type IVA Humerus Resection		Endoprosthesis	No
25	20	Osteosarcoma	Left Proximal Humerus	Type IA Humerus Resection		Endoprosthesis	No
26	23	Osteosarcoma	Left Proximal Humerus	Type IIB Humerus Resection		Endoprosthesis	No
27	55	Metastatic Renal Cell Carcinoma	Left Proximal Humerus	Type IA Humerus Resection		Endoprosthesis	No
28	50	Giant Cell Tumor	Right Proximal Humerus	Type IA Humerus Resection		Endoprosthesis	No
29	6	EW Sarcoma	Left Scapula	Type III Scapula Resection		No reconstruction	No
30	22	Ewing Sarcoma	Right Scapula	Type III Scapula Resection		No reconstruction	No
31	20	Osteosarcoma	Right Proximal Humerus	Type IIA Humerus Resection		Endoprosthesis	Yes
32	40	Osteosarcoma	Right Proximal Humerus	Type IA Humerus Resection		Endoprosthesis	No

Surgical resection margins were wide (> 2 mm) in 28 patients, close (< 2 mm) in three patients and positive in one patient. Thirty cases exhibited no recurrence while two patients developed local recurrence (osteosarcoma (*n* = 1) and Ewing sarcoma (*n* = 1)). In both these patients, the local recurrence was part of systemic recurrence and both patients died from the disease.

Twenty-five patients were reconstructed using endoprosthesis, 21 patients using GMRS stryker Howmedica (Stryker Howmedica, Mahwah, NJ, USA), four patients using a custom-made prosthesis from Stanmore (Stanmore, Elstree, England), two of them custom-made scapula, three patients using osteoarticular allograft, and four with no reconstruction. One patient developed stem loosening while two patients had a deep infection that mandated debridement and hospital admission; one patient had osteoarticular allograft failure and was revised using custom endoprosthesis. From a functional point of view, all patients lost overhead activity except one with osteoarticular allograft and preservation of the rotator cuff. In this case, surgery was done for massive osteolysis of the proximal humerus (Gorham’s disease). For all patients, the average MSTS functional score was 83 (range, 77–88%).

**Fig. 14:**
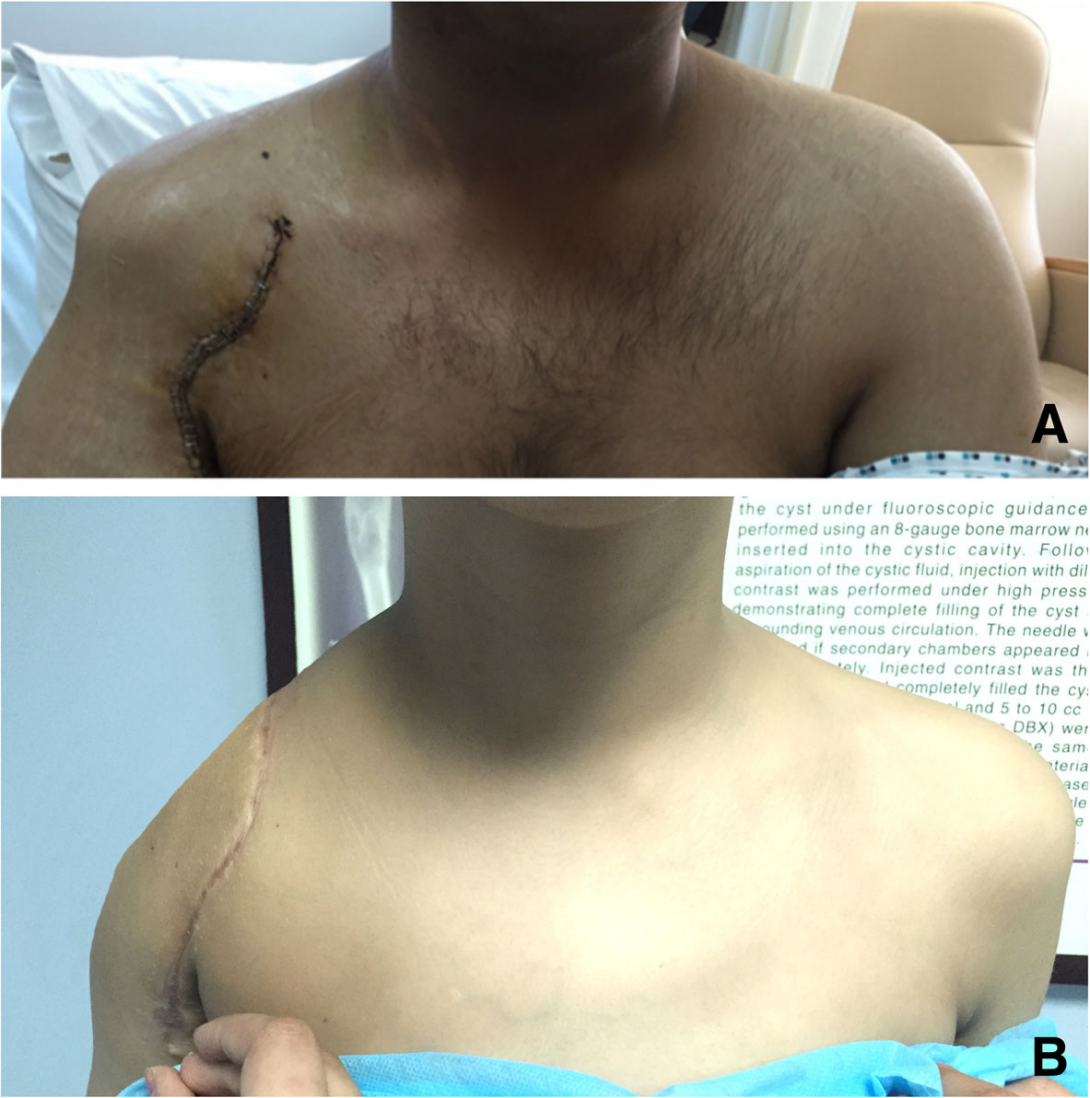
**a** Shoulder resection with preservation of normal shoulder contour. **b** Shoulder resection with previously described techniques

The appearance of patients who received extra-articular resection revealed preservation of the normal contour of the shoulder (Fig. 14a) in contradiction to the appearance using the previously published techniques (Fig. 14b). Rehabilitation of all patients using our modified surgical techniques was made easier by permitting the patient to do full elbow function immediately after surgery giving the fact that his coracobrachialis muscle was kept undetached from its insertion during surgery. The two patients with scapular prosthesis show better cosmetic appearance when compared to the other 4 who did not receive endoprosthetic reconstruction; however, the functional outcome was more or less the same.

## Discussion

All previously reported shoulder girdle surgical series consistently allow both humeral and scapular resections in the same classification system [2, 3, 6, 7]. This is in defiance to common wisdom as these are two different bones with different anatomical specifications, surgical approach, and reconstruction methodology. Our new classification is the first classification system to address the scapula and the humerus separately.

The classification system proposed by Malawer [2] has several limitations. Firstly, there is a numerical discontinuity which makes recalling this classification difficult, for example, proximal humerus is type I, scapular resection is type II, followed by proximal humerus resection with glenoid as type V. In addition, this classification system did not address the resection of the whole humerus which was performed on two patients in this study.

Furthermore, the Malawer classification assigned types A and B (deltoid preserving vs. sacrificing) to the scapular resection, while anatomically the deltoid muscle will not be part of any attempted scapular resection [1].

For the Musculoskeletal Tumor Society classification [3], a regional name from S1 to S5 is given for each part of the proximal humerus and the scapula. For example, the diaphysis of the humerus is S5, the diaphseometaphyseal area is S4, the humeral head is S3, the glenoid and scapular neck are S2, and the rest of the scapula is S1. Therefore, a Type II humeral resection as defined by our study would be written as S5S4S3S2 or S4S3S2, which can be impractical. In addition, this classification system also does not address the whole humeral resection.

The previous surgical techniques described in the literature for intraarticular proximal humerus resection unjustifiably detach the conjoint tendon from coracoid when it can usually be left and retracted [2, 5]. For extra-articular resections, the unjustifiable sacrifice of the coracoid, acromion, and the distal third of the clavicle has been common practice and published in previous literature surgical techniques [1] with no oncological reasoning or superiority to our approach where these structures are preserved. This could result in superior aesthetic outcomes.

## Conclusion

The proposed classification system is easy to apply and implement for all cases that are likely to be faced by the orthopedic surgeon. It is also relatively easy to recall which could allow widespread use. The modifications in the surgical techniques in our experience have enabled patients to be rehabilitated faster, improving the appearance of shoulder contour which is cosmetically superior and allows better clothes fitting, comparable to the unaffected side which helps maintain a normal body image.

## Data Availability

No pre-existing data and material was used for this study.
